# Bilateral precalcaneal congenital fibrolipomatous hamartoma

**DOI:** 10.1007/s00247-026-06570-w

**Published:** 2026-03-12

**Authors:** Carolina Ávila de Almeida, Clarissa Canella, Elazir Barbosa Mota Di Puglia

**Affiliations:** 1 Department of Pediatric Radiology of Universidade Federal do Rio de Janeiro, Rua Rua Bruno Lobo, nº 50, Cidade Universitária, Rio de Janeiro, RJ 21941-91 Brazil; 2 Fluminense Federal University, Niterói, Brazil


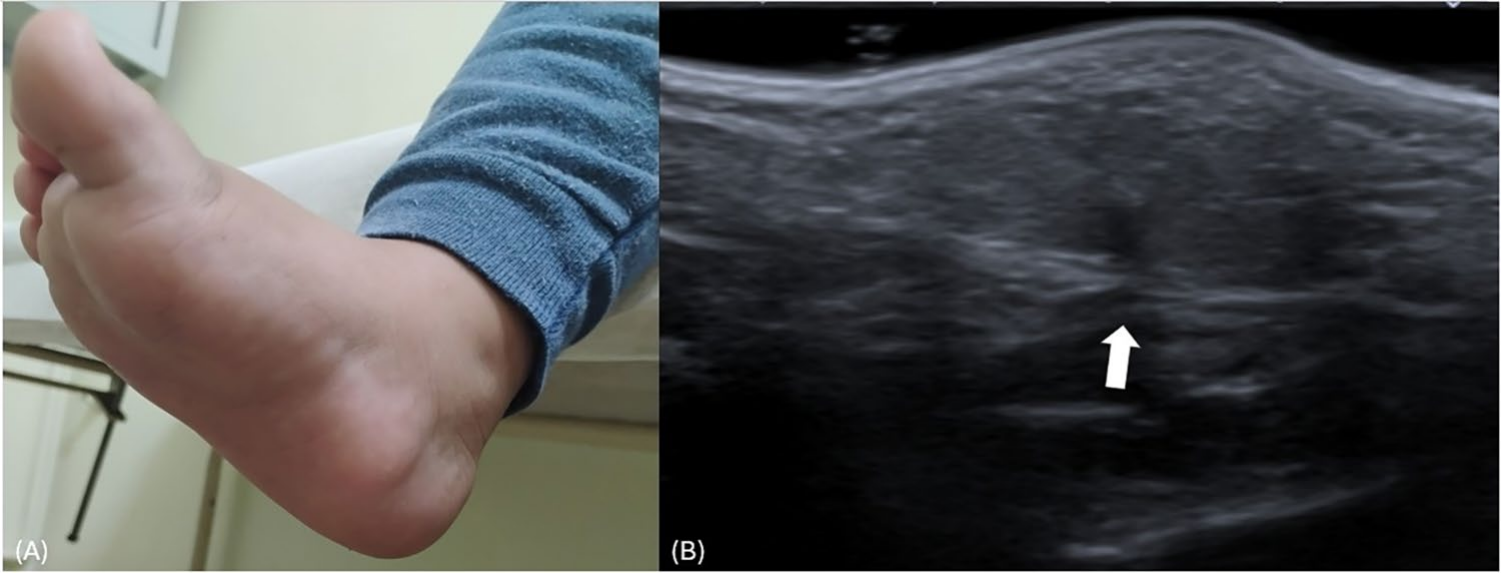


A 2-year-old girl presents with bilateral soft-tissue nodules along the medial precalcaneal plantar regions of the heels, present since birth and enlarging proportionally with growth. Clinical photograph (A) demonstrates symmetric, skin-colored, nontender nodules without overlying skin changes (arrows). Ultrasound was performed for further characterization. Longitudinal ultrasound image (B) shows a homogeneous, slightly hyperechoic subcutaneous lesion without infiltration of the underlying fascia or deeper structures (arrow). No internal vascularity is detected on Doppler imaging. Imaging findings are consistent with precalcaneal congenital fibrolipomatous hamartomas, a benign condition characterized by localized proliferation of mature adipose tissue within the subcutaneous layer. Recognition of this typical clinical and sonographic appearance allows confident diagnosis and prevents unnecessary biopsy or surgical intervention.

## Data Availability

No datasets were generated or analysed during the current study.

